# A Case of Protracted Labor

**Published:** 1896-11

**Authors:** J. N. Upshur

**Affiliations:** Richmond, Va., Professor Practice of Medicine Medical College of Virginia; 210 West Grace Street


For the Texas Medical Journal.

### A CASE OF PROTRACTED LABOR.

### Rupture of the Uterine Wall—Concealed Hemorrhage Subsequent Extensive Sepsis and Mural Abscess, etc., Ending in Recovery.

BY J. N. UPSHUR, M. D., RICHMOND, VA.,
Professor Practice of Medicine Medical College of Virginia.

[Read before Richmond Academy of Medicine and Surgery, October 13, 1896.]

MRS. H., æt. 40, of good health prior to two months before delivery, when she had attack of dysentery. I saw her at that time. She had a recurrence of the attack twice, but treated herself with the same remedies prescribed by me during the first attack. With this exception, her pregnacy had been an uneventful one.

Labor came on suddenly at 7 p. m. September 11th—pains so active and strong that I was twice sent for within an hour. When I reached my patient at 8 p. m., she looked pallid and showed evidence of extreme suffering—pains were strong and regular, coming on every few minutes. Digital examination revealed a cervix *very rigid*, thick and about two inches long, through which could be felt the vertex presenting in the first position. The pains, though violent and causing much suffering, made little impression in dilating the rigid cervix. To procure rest and relaxation of the cervix, about 12 p. m., I administered a hypodermic of sulphate morphia, grain one-fourth, sulphate atropiæ, grains $\frac{1}{150}$. This procured her some respite from suffering for three hours, when pains again became active. Cervix had slightly softened. She was now given chloroform, but only with the effect of stopping pains. A second hypodermic was given at 7 p. m., and I left the patient for three hours.

On my return at 11 a. m., I found cervix relaxed and dilatable, and pains active and regular. Patient looked palid, with feeble pulse, and had clammy skin. But that she had had three exhaustive attacks of bowel trouble and a wearing labor from the beginning, I should have suspected concealed hemorrhage. Chloroform was again administered, and she was normally delivered at 12-45 of a still-born male infant weighing nine pounds. She had last felt the motion of the child just prior to the onset of labor. There was delay in the delivery of the placenta, and the uterus was in a state of complete inertia. Examination showed the placenta detached, and it was easily removed; introduction of the hand being followed on delivery by a double handful of coagula. It was evident that partial detachment of the placenta had taken place prior to the birth of the child.

The uterus remaining obstinately relaxed, the hand was passed into the cavity, which presented a feeling of a ragged bag, while on the right of the uterine wall, half way between its anterior and lateral portion, could be felt in incomplete *rupture* of the uterine wall in its long diameter, about three inches long, and extending four-fifths of the thickness of the uterine wall, so that a narrow escape had been made from opening the peritoneal cavity. After administration of sulphate strychnia grains $\frac{1}{20}$, and fluid extract ergot (Squibbs) ℨv, with introduction of ice into the cavity, the womb was induced to contract finely. I would have preferred a hot salt solution, but it was not available, and only a septic syringe with which to use it, if it had been. The patient reacted well. Having always had a free secretion of milk, tincture of camphor was ordered applied to the breasts, beginning a few hours after delivery, extending its application well into the axillæ. This proved efficient, and no milk was secreted.

On the second and third days after delivery, temperature only rose to 99.4°F. On fourth day it reached 100°F. Lochia profuse, offensive, of the color of dirty dish water. Free hot douche of hot water and borax was ordered thrice daily.

But on the morning of the fifth day, finding no improvement except temperature slightly subnormal (98)°F.), the uterus was washed out freely with hot salt solution. Uterus was found above the pubis, and spongy. Ordered tincture chloride iron, ergot and nux vomica in full doses every four hours.

On sixth day found little change in lochia, and that seventeen napkins had been used in previous twenty-four hours; pulse fair at 90-100; temperature as on day before. Curette (sharp) was used, and a number of dirty shreds of sloughing tissue were removed.

On seventh day, patient expressed herself as feeling better; pulse and temperature same as on day previous. Curette was again used, and large pieces of sloughing tissue were removed; curette gave way suddenly, giving sensation of entering a cavity, and was followed by a discharge of an ounce or more of thick, dirty-colored stinking pus. I believe the rupture had healed on the surface, and an abscess had developed at bottom. The curette was freely used until discharge was slightly stained with blood. Half gallon of salt solution was run through the uterine cavity till it came away clean and odorless; patient expressed herself as feeling much more comfortable.

On eighth day, pulse and temperature same; ten napkins used; curette again applied; no shreds. Washed out with peroxide of hydrogen, followed by salt solution, and lightly packed with iodoform gauze.

Twenty-four hours after delivery, the patient complained of severe headache, located chiefly in the occiput. This was relieved by two doses of phenacetin of gr. v. each, but returned when the drug wore off. Subsequently benefited by sodii bromidi gr. xx, and caffeine gr. ss. Patient had a comfortable night, has some appetite, expressed herself as free of headache, and feeling much better; said headache returned after treatment of womb. She has taken on two days full doses of quinine, but I could detect no benefit from its exhibition. Bowels have been moved on alternate days by exhibition of compound senna powder. Tongue has been clean, but pallid from beginning.

On ninth day, eleven napkins in last twenty-fours hour; temperature, 100°F.; pulse, 108; had a good night, but sweated freely; appetite fair; discharge very little offensive; curette used; ℨj of pus discharged from uterine cavity; came from site of rupture; uterus reduced decidedly in size; washed out freely with peroxide hydrogen, one-fourth to three-fourths hot water, followed by salt solution. Patient complained of violent frontal headache during the dressing, which passed off when completed; light packing of iodoform gauze applied.

Tenth day, eight napkins; pulse, 100; temperature, 99.4°; no odor in discharge; on removing gauze, ℨss pus flowed away; uterus cannot be felt above pubis; measured three inches with probe; swabbed out with pure peroxide and replaced with gauze; complained of severe frontal headache during the dressing, which subsided when it was completed.

Eleventh day, five napkins; temperature, 99.2°; pulse, 100; on removing gauze, ℨss pus; no sweating.

Thirteenth day, temperature, 99.4°; pulse, 100; no sweating.

Fourteenth day, pulse, 96; temperature, 99°F.; no flow.

Sixteenth day, pulse, 100; temperature, 98.5°; convalescence established; patient sat up an hour and a quarter.

Twenty-eighth day, patient dressed and moving about her room; says she is absolutely comfortable; no pain or weakness in back or elsewhere, except a little unsteady on her feet.

*Remarks.*—The above case presents first in interest, the *condition of the cervix* at the beginning of labor, so *hard and resistant* as to give the impression of previous disease, which had not been the case, except a slight cervical catarrh, for which I had treated her prior to her sixth confinement, which had been rapid and uneventful. Pains seemed absolutely inefficient in producing dilatations until after the administration of morphine—the resistance in spite of such strong contractions being responsible for the subsequent complications. I am sure that the patient's pallor was due to concealed hemorrhage from premature partial separation of the placenta, not appreciated at the time because of my knowledge of three attacks of dysentery in the previous two months and the suffering she had borne incident to the violent uterine action, ineffective and depressant. The inertia of the womb was simply muscular tire.

The rupture of the uterine wall in its long axis is of interest, because the almost invariable rule is that when rupture occurs, it is in the transverse direction. "Boudl reported nineteen cases in 40,614 labors occurring in nine years in the Lying-in Hospital in Vienna. Jolly, in Paris, found 230 cases in 782,-741, excluding from his list lacerations of the cervix. Harris estimates one case in 4,000 labors. Lusk found forty-seven deaths from this cause in New York between 1867 and 1875, inclusive, or one death in six thousand labors."—(*Lusk's Obstetrics*, page 603.) I had a case of complete rupture due to an after-coming hydrocephalic head—patient dying five hours after delivery.

Hagenberger estimated the mortality at 95 per cent.; C. Braun at 89 per cent. (*Lusk*, page 603). The above facts show how rare is this complication, even though incomplete.

Another point of interest is the prevention absolutely of the milk formation by the *early* and *repeated* use of tincture of camphor in a woman who had in six previous confinements had an abundance. A long experience has proven no remedy better than the camphor in my hands. The fact that only twice did the temperature reach 100°F. in a uterus so septic as I have described, is remarkable, and the efficient action in the direction of cure of free curettage, douching with salt solution and application of hydrogen dioxide in stopping suppuration, evidences the great value of this therapeutic method. The violent reflex headache coming on during the dressing and disappearing when it was completed, is of interest, and shows plainly that the violent headache coming on twenty-four hours after labor, was also reflex in character.

I deem myself most fortunate to have had such a satisfactory results in this case, and place it on record for the instruction and encouragement of my professional brethren.

210 *West Grace Street.*

